# The healthcare costs of intoxicated patients who survive ICU admission are higher than non-intoxicated ICU patients: a retrospective study combining healthcare insurance data and data from a Dutch national quality registry

**DOI:** 10.1186/s12873-019-0224-7

**Published:** 2019-01-11

**Authors:** Ilse van Beusekom, Ferishta Bakhshi-Raiez, Nicolette F. de Keizer, Dylan W. de Lange

**Affiliations:** 1 Department of Medical Informatics, Amsterdam Public Health research institute, Amsterdam University Medical Center, University of Amsterdam, Amsterdam, The Netherlands; 2 National Intensive Care Evaluation (NICE) Foundation, Amsterdam, the Netherlands; 3 Dutch Poisons Information Center (DPIC), University Medical Center, University Utrecht, Utrecht, the Netherlands; 4 Department of Intensive Care, University Medical Center, University Utrecht, Utrecht, the Netherlands

**Keywords:** Costs, Intoxication, Poisoning, ICU, Survival, Comorbidity

## Abstract

**Background:**

The aim of this study was to describe the healthcare costs of intoxicated ICU patients in the year before and the year after ICU admission, and to compare their healthcare costs with non-intoxicated ICU patients and a population based control group.

**Methods:**

We conducted a retrospective cohort study, combining a national health insurance claims database and a national quality registry database for ICUs. Claims data in the timeframe 2012 until 2014 were combined with the clinical data of patients who had been admitted to an ICU during 2013. Three study populations were compared and matched according to socioeconomic status, type of admission, age and gender: an “ICU population”, an “intoxication population” and a “control population” (who had never been on the ICU).

**Results:**

2591 individual “intoxicated ICU patients” were compared to 2577 general “ICU patients” and 2591 patients from the “control population”. The median and interquartile ranges (IQR) healthcare costs per day alive for the “intoxicated ICU patients” were higher during the year before ICU admission (€20.3 (IQR €3.6–€76.4)) and the year after ICU admission (€23.9 (IQR €5.1–€82.4)) compared to the ICU population (€6.1 (IQR €0.9–€29.3) and €13.6 (IQR €3.3–€54.9) respectively) and a general control population (€1.1 (IQR €0.3–€4.6) and €1.1 (IQR €0.4–€4.9) respectively). The healthcare associated costs in intoxicated ICU patients were correlated with the number of chronic conditions present prior ICU admission (*p* < 0.0001).

**Conclusions:**

Intoxicated patients admitted to the ICU had in the year before and after ICU admission much higher median healthcare costs per day alive compared to other ICU patients and a general population control group. Healthcare costs are greatly influenced by the number of psychiatric and other chronic conditions of these intoxicated patients.

**Electronic supplementary material:**

The online version of this article (10.1186/s12873-019-0224-7) contains supplementary material, which is available to authorized users.

## Background

It has been suggested that Intensive care Unit (ICU) survivors often suffer from long-term sequelae that may significantly increase healthcare costs to society [1]. Indeed, it has been shown that over a 2-year period, patients admitted to the ICU with sepsis have monthly healthcare expenditures three times higher than prior to their ICU admission [1, 2]. Depending on region and healthcare system it is estimated that between 2.7 and 40% of patients seen in the emergency room are subsequently admitted to the ICU and between 3.4 to 14% of ICU admissions are admitted for intoxications [3–5].

The majority of intoxication in developed countries are accidental [6]. For example, in the Netherlands half of the information requests to the Dutch Poison Information Center (DPIC) involve human medications, in 14% patients are exposed to house hold products, 12% to food additives or drugs of abuse [7]. In 33% of the intoxications small children are involved (age 0–4 years). These intoxications are almost all accidental. In 40% of the inquiries to the DPIC involve adults of ≥18 years old. In these adult patients only half of the intoxications is accidental, the other half is often with a suicidal intent. Some of the accidental intoxications do not need medical treatment and are not referred to a hospital. More severe intoxications (both accidental or intentional) in adults are treated in the emergency department and many of those patients are admitted to the hospital (or even to the Intensive Care Unit (ICU) [8, 9]. Many of the intoxicated ICU patients have a short length of stay on the ICU and the hospital and long-term mortality are relatively low [10]. This suggests that cost/effectiveness ratio, which is defined by the cost of treatment divided by the expected years alive, is supposedly very good for intoxicated patients. However, long-term sequelae are often ignored in these analyses.

Therefore, the aim of this study is to evaluate the healthcare-related costs of intoxicated patients in the year before their ICU admission in comparison to the costs in the year after their ICU admission and to compare these costs to that of non-intoxicated ICU patients and the general population.

## Materials and methods

### Study design

We performed a retrospective cohort study using the Dutch National Intensive Care Evaluation (NICE) registry [11]. The NICE registry is a national quality registry in which all Dutch ICUs participate and collect clinical, demographic, physiologic, and outcome data from all admitted patients. This includes all variables required to quantify the severity of illness and to calculate case-mix adjusted mortality risks according to the Acute Physiology and Chronic Health Evaluation (APACHE) IV model [12].

We combined data from the NICE registry and the insurance claims database (Vektis) [13]. Health insurance is obligatory for all Dutch citizens and 99% have private healthcare insurance. Vektis is an insurance claims database where all reimbursements of healthcare costs are registered [14]. Although insurance claims information of patients is aggregated in the Vektis database Dutch patients do not directly contact nor reimburse the Vektis database.

### Subjects

All patients from the NICE registry aged ≥18 years during the year of ICU admission, admitted to an ICU during 2013, and discharged from the hospital before January 1st 2014 were included in the NICE registry subset.

Patients from the Vektis database were identified as ICU patients when they had a claim for an ICU day in the year 2013. All patients of 18 years or older during the year of ICU admission were included in the ICU-subset of the Vektis database.

Based on this ICU-subset a comparable population was extracted from the registered inhabitants of the Netherlands in the Vektis database. This population-based control group was weighted on the combination of the variables gender, age and socio-economic status (SES) and had no claims for ICU care during 2013. For every ICU patient in the Vektis ICU-subset, one control patient was selected. If one of the three variables used for weighting was missing, such a control patient was not selected.

### Setting

The year before ICU admission is defined as January 1st 2012 until December 31st 2012, the year of ICU admission was defined as January 1st 2013 until December 31st 2013 and the year after ICU admission is defined as January 1st 2014 until December 31st 2014.

### Linking process

Clinical data from the NICE database were anonymously linked to cost data from the Vektis database using a deterministic linkage algorithm [15]. The process of linking the NICE database and the Vektis database is published previously [2].

### Matching

After linking the two databases (Vektis and NICE) patients who were admitted to the ICU with an intoxication were selected using the APACHE IV admission diagnosis of intoxication in the NICE registry (see Additional file 1: Table S1 for definitions). These patients made up the “intoxication population”. The latter was was matched 1:1 with patients in the combined database who were admitted to the ICU for reasons other than intoxication (the so-called “ICU population”). Matching was done based upon age, gender, admission type and SES. An ICU patient could only be matched if there were no missing items used for matching. The intoxication population was matched 1:1 with people in the combined database not admitted to the ICU. Matching for this “control population” was done based upon age, gender and SES.

### Comorbidities and chronic diseases

Healthcare costs are related to chronic conditions requiring pharmacological and other medical treatments. We determined the underlying medical conditions present at admission to the ICU from the APACHE IV severity of illness model. Additionally we looked at proxies for underlying medical conditions and concomitant diseases from the Vektis database. For example, patients with reimbursed costs for diabetic medications were attributed a diabetes comorbidity (see Additional file 1: Table S2). Costs per day were analyzed in relation to the number of underlying medical conditions, as described previously [2].

### Statistical analysis

The primary outcomes of this study are the healthcare costs per day alive of intoxicated patients in comparison to the healthcare costs of the ICU population and the control population, during the year before ICU admission, the year of ICU admission and the year after ICU admission.

The healthcare costs are only available as a total sum per person per calendar-year. We converted the total costs per calendar-year into healthcare costs per day alive, presented in Euros. ICU patients who did not survive their ICU admission were excluded from all analyses as these patients have by definition no (costs per) day alive after IC admission.

Descriptive statistics were used to characterize the demographic data of the study populations. Mean and standard deviation (SD) are given for normally distributed data, median and IQR are provided for non-normally distributed data, numbers and proportions are used to present categorical data.

General linear modelling was used to estimate the cohort effect on the healthcare costs per day alive during the year before ICU admission, on the healthcare cost per day alive during the year of ICU admission and on the healthcare cost per day alive during the year after ICU admission. As healthcare costs per day alive were skewed to the right the natural logarithm of the healthcare costs per day alive was used. Because of multiple comparisons a more stringent *p*-value of < 0.01 was considered to indicate a statistically significant difference.

### Subgroups analyses

Previous research has shown that healthcare costs are higher in the last 120 days prior to death [16]. Therefore, a survival curve was constructed to gain insight in the long-term mortality of all three study populations. For the survival analyses, the period at risk starts at January1^st^, 2013.

Analyses were performed for the total study population, for a subgroup which died during 2013, for a subgroup which died during 2014, and for a subgroup which survived the entire study period. Additionally, for all three-study populations we created subgroups based on the number of chronic medical conditions.

The intoxication population and the ICU population were divided into subgroups based on the APACHE IV predicted mortality; i.e. low-risk (predicted mortality < 30%), medium-risk (predicted mortality ≥30 and < 70%) and high-risk (predicted mortality ≥70%). Analyses regarding the APACHE IV predicted mortality were only preformed for ICU admissions which met the APACHE IV inclusion criteria. Furthermore, we grouped the intoxication population and the ICU population by length of stay (LOS) of their first ICU admission. Groups were made of patients with a LOS of < 2 days and patients with a LOS of ≥2 days.

### Ethics

The Medical Ethics Committee of the Academic Medical Center approved this study (number W18_010 # 18.021).

## Results

**Fig. 1:**
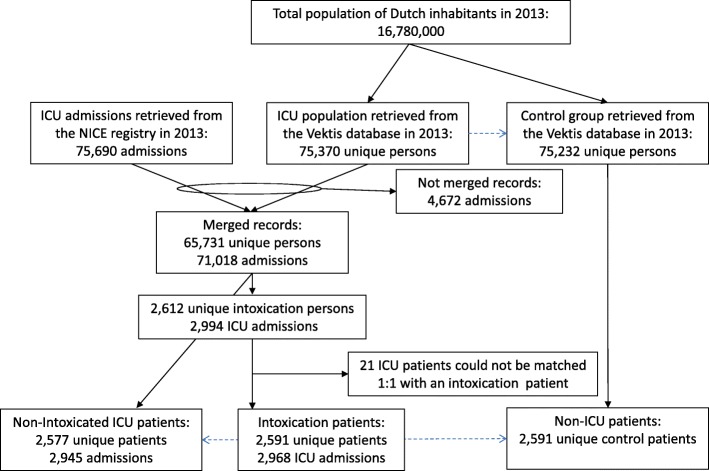
Flow of patients and the linking process

The final dataset included 2591 patients admitted to the ICU for intoxication. These 2591 patients had 2968 ICU admissions in 2013. Intoxication was the underlying reason for 95.8% (*n* = 2843) of admissions whereas 4.2% (*n* = 125) were admitted for reasons other than an intoxication. Based on the intoxication population, 2577 ICU patients were matched 1:1. Some patients (*n* = 14 ICU patients) could not be matched 1:1 due to missing information. The ICU patients were (re)admitted 2945 times to the ICU. Finally, 2591 control persons were matched 1:1 with the intoxication population. Figure 1 gives an overview of the data linkage and data matching process.

Table 1 gives an overview of the characteristics of the three study populations. The mortality of the “ICU population” was significantly higher than that of the “intoxication population” (*p* < 0.0001). (Additional file 1: Figure S1) illustrates survival curves for the intoxication, ICU and control population.

**Table 1:** Characteristics of the intoxication patients, the ICU population and the general population control patients

Variable	Intoxication patients (*n* = 2591)	Other ICU patients (*n* = 2577)	Control population (*n* = 2591)
Male (n %)	1185 (45.7%)	1179 (45.8%)	1185 (45.7%)
Age (median IQR)	45 (32; 55)	45 (32; 55)	45 (32; 55)
SES (median IQR)	0.1 (−0.8; 0.7)	0.1 (−0.8; 0.7)	0.1 (− 0.8; 0.7)
Died during 2013 (n %)	141	488	17
Died during 2014 (n %)	107	96	11
Characteristics of the first (intoxication related) ICU admission			
Admission type			
• Medical	2563	2563	
• Planned surgery	6	6	
• Emergency surgery	8	8	
• Missing	14	–	
APACHE IV score^a^	38 (24; 62)	49 (31; 76)	
Length of ICU stay (days, median, IQR)	0.8 (0.5; 1.3)	1.7 (0.8; 3.6)	
Length of hospital stay (days, median, IQR)	1 (1; 3)	8 (4; 15)	
Mechanical ventilation	537	1016	
Subgroups of intoxications			
• Alcohol	277	–	
• Analgesics	110	–	
• Antidepressant	282	–	
• Street drug	357	–	
• Sedatives	836	–	
• Poisoning	11	–	
• Other	364	–	
• Combination	354	–	
Acute diagnosis			
• Cardiopulmonary resuscitation	26	218	–
• Burns	2	13	–
• Cardiac dysrhythmia	97	238	–
• GI bleeding	6	79	–
• CVA	14	118	–
• Intracranial mass effect	8	113	–
• Sepsis	8	316	–
• OHCA	22	168	–
• SAH	0	60	–
• Trauma	54	287	–

^a^only calculated for patients which met the APACHE IV inclusion criteria, which was *n* = 2456 for intoxication patients group and *n* = 2392 for ICU patients group

**Fig. 2:**
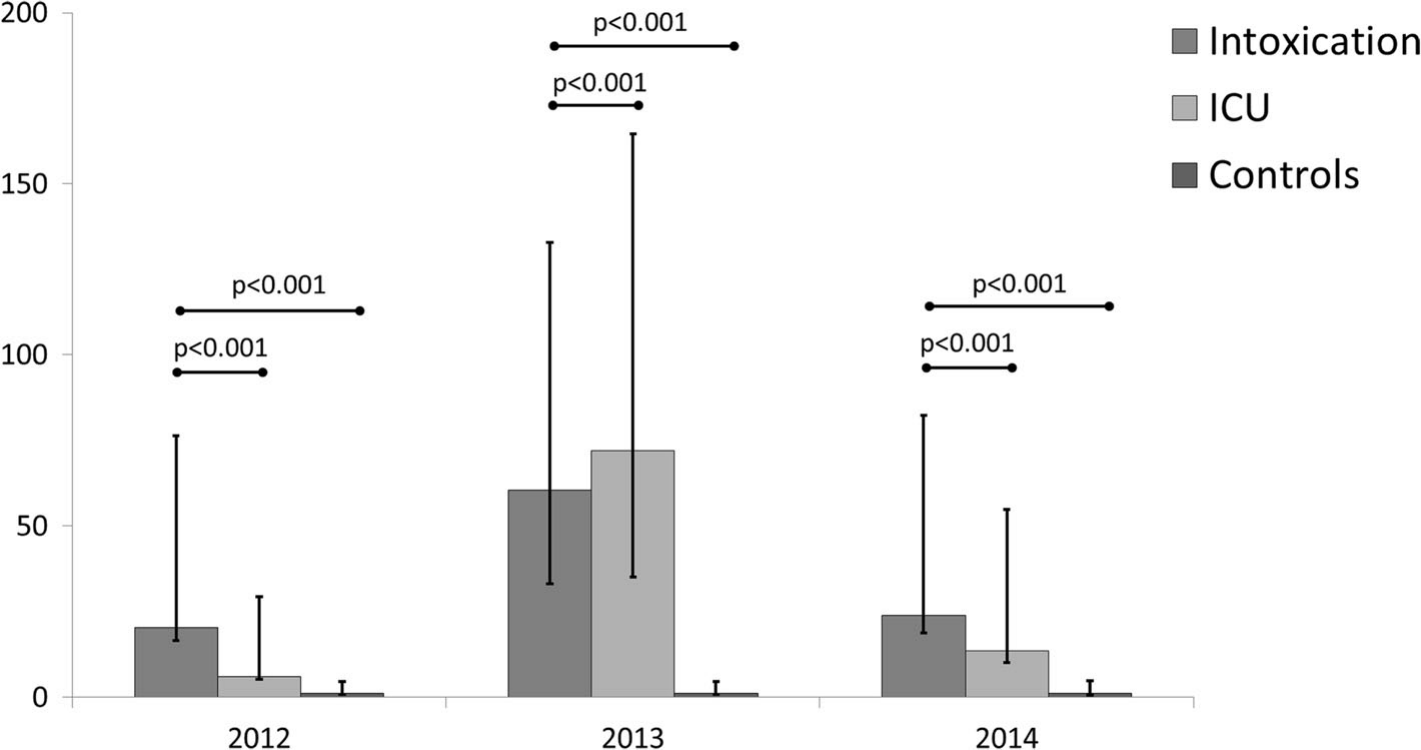
The costs of intoxicated, ICU and control subjects. The median costs (in euro’s and the 25th and 75th percentile) per day alive for those patients that survived all 3 years. Intoxicated patients admitted to the ICU (*n* = 2343), ICU patients admitted for other reasons than intoxication (*n* = 1993) and control patients who were never admitted to the ICU during the study period (*n* = 2563)

The healthcare costs per day alive of the intoxication group survivors were compared to the ICU group survivors and to the control group survivors. The healthcare costs of the intoxication population were higher during the year before ICU admission (€20.3 (IQR €3.6–€76.4)), compared to those of the ICU population (€6.1 (IQR €0.9–€29.3)) (*p* < 0.0001) and compared to those of the control population (€1.1 (IQR €0.3–€4.6)) (*p* < 0.0001). During the year of ICU admission the costs per day alive for the intoxication population were €60.5 (IQR €27.4–€132.8) in comparison to the ICU population (€72.1 (IQR €37.0–€164.6), *p* < 0.0001). In the year after ICU admission the costs per day alive for the intoxication population were €23.9 (IQR €5.1; €82.4) in comparison to the ICU population (€13.6 (IQR €3.3–€54.9), *p* < 0.0001) and in comparison to the control population (€1.1 (IQR €0.4–€4.9), *p* < 0.0001) (see Fig. 2).

Those within the intoxication group surviving all three years who were intoxicated with “sedatives” had the highest median healthcare costs per day alive during the total study period (*p* < 0.0001). An overview of the cost per day alive for various intoxication groups is provided in the (Additional file 1: Figure S2).

Fifty-three percent (1389/2591) of patients in the intoxication group had one or more underlying medical conditions at the time of their ICU admission, compared to 40.3% (1038/2577) in the ICU group. Approximately 19% (489/2591) of those in the control group had one or more medical condition (see Additional file 1: Table S2). The most prevalent accompanying conditions in the intoxication population were depression (*n* = 599), psychoses, Alzheimer’s disease and addictions (*n* = 357) and high cholesterol (*n* = 151). More details are provided in the Additional file 1: Tables S2, S3 and S4.

**Fig. 3:**
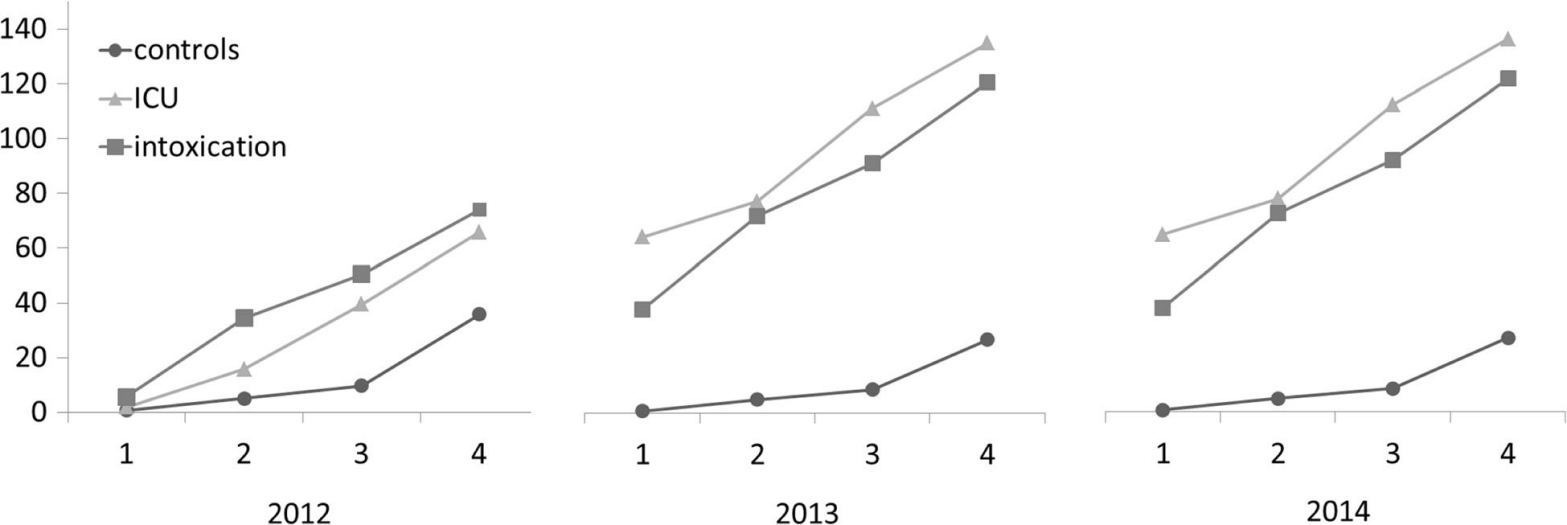
The costs per day alive in relation to the number of comorbidities. Costs (in euro’s) per day alive (on the y-axis) in relation to the cumulative number of conditions and comorbidities of intoxicated patients (squares, *n* = 2591), ICU patients (triangles, *n* = 2577) and control patients (circles, *n* = 2591) who never were admitted to the ICU (on the x-axis. Depicted is the year prior to ICU admission (2012), the year of ICU admission (2013) and the year after ICU admission (2014). An asteriks (*) denotes a statistical difference (*p* < 0.0001)

The costs per day alive in relation to the number of underlying medical conditions is depicted in Fig. 3. The intoxication population showed an increase in healthcare costs per day alive in relation to the number of comorbidities. In the year after ICU, people with a greater number of chronic conditions had higher healthcare costs per day as well. During the year after ICU admission, the effect of number of chronic conditions on the healthcare costs was the same within the ICU population and the intoxication population (*p*-value for interaction: *p* = 0.44).

The costs in relation to severity of disease has been depicted in Additional file 1: Figure S3.

## Discussion

The primary finding of this study is that patients admitted to the ICU for an acute intoxication have higher healthcare costs per day alive in the year prior to their admission, compared to non-intoxicated ICU patients or matched controls. Furthermore, healthcare costs per day alive remain elevated in the year following their admission.

Previous studies on the costs of intoxicated patients admitted to the ICU only focused on direct ICU-associated or hospital-associated expenditures [17–25]. Obviously, the costs per ICU treatment depends on country, region, healthcare system and type of intoxication. Only very few studies have looked at the healthcare associated costs beyond hospital admission. Indeed, many of these studies identified this as a limitation of their studies as many of the costs are associated with newly instigated or intensified treatment for underlining psychiatric illnesses or newly acquired organ dysfunction [26]. For example, in a nationwide, Japanese study 17 per 100,000 inhabitants were admitted to acute care hospitals for accidental or intentional intoxications. More than 60% had been assessed by a psychiatrist in the 90 days preceding their intoxication [27]. After hospital discharge approximately 12% were transferred to a psychiatric department. This suggests that a proportion of healthcare costs are being made outside the acute care setting. One of the few studies that assessed patients 6-months beyond discharge found that intoxications had the lowest cost/effectiveness ratio, defined as the cost of treatment divided by the expected years alive [28]. If this life expectancy is corrected by the health-related quality of life (HRQoL), then the costs of ICU treatment would be 620 United States dollars per quality adjusted life year (QALY). However, the sample of intoxicated patients in that study was very small (*n* = 23) and the healthcare costs after hospital discharge were, again, not incorporated [28]. We, unfortunately, could not calculate QALYs as HRQoL was not in our database. However, from a small study of Dutch survivors of intoxications we know that the HRQoL was statistically significantly lower than that of the general Dutch population [29]. Moreover, 25% of these patients had very low HRQoL. This suggests that the cost/effectiveness ratio of the Dutch intoxicated patients surviving ICU admission may be poorer despite reasonable survival rates in this population.

We have shown that the healthcare related costs for intoxicated patients were high(er) in the year prior to ICU admission. This is a well-known phenomenon in many other subgroups. In a general ICU population factors present before ICU admission, such as comorbidities and pre-ICU hospitalizations, were stronger predictors of hospital resource use than acute severity of illness [30]. In this latter study the Simplified Acute Physiology Score II (SAPS II) was used whereas the APACHE IV model was used in the present study. The APACHE IV model includes more chronic factors compared to the SAPS II model, and for this reason we would expect that ICU patients within the highest mortality risk group would consume the most healthcare resources during the year before and the year after ICU admission. Indeed, the patients in the “intoxication subgroup” had the highest prevalence of comorbidities or accompanying conditions in comparison to the “other ICU patients” and the “control subgroup”.

We have also shown that intoxicated patients have more chronic conditions prior to ICU admission than other patients admitted to the ICU. This surplus of accompanying conditions and comorbidities is driven by neuropsychiatric disorders in this particular population. Stratifying the healthcare costs per day alive by the amount of chronic conditions showed great deviation from the median healthcare cost per day alive, indicating that those factors largely contribute to the healthcare costs.

Our study has several limitations. First, the total costs per patient were only known per calendar-year. It is unclear which aspects of healthcare were most utilized or which aspects were most expensive. For example, patients admitted in December will have a spill over of costs in the next calendar year and, vice versa, such patients will be cheaper during the first months of the year of ICU admission. This might exaggerate the difference between the costs in the year prior to ICU admission in comparison to the year after ICU admission. Second, because costs were provided as total costs per year we could not dissect which components of care (e.g. mechanical ventilation, hemodialysis, salaries of healthcare workers, laboratory assessments, etc.) were important drivers of the costs. However, previous research has shown that costs of human resources made up an important part of the total costs [6]. Last, we did not have a control group of intoxicated patients that was not admitted to the ICU. These patients could not be identified in both databases. We are, therefore, unaware of the increase in costs per day alive of those patients. We can only speculate that these patients (admitted to other wards of the hospital) also have intensified (psychiatric) care after their intentional intoxication.

Despite these limitations, the linkage between the national health insurance claims database and the national clinical ICU registry, covering almost the entire country, provides valuable insight in the healthcare utilization of intoxicated patients who are admitted to the ICU.

## Conclusions

We showed that intoxicated people who were admitted to an ICU had higher healthcare costs per day alive compared to an other ICU population and a control population. The difference in healthcare costs is already present in the year before ICU admission and continues during the year after discharge. The healthcare costs before and after ICU admission are greatly influenced by the chronic and psychiatric conditions of patients.

## Additional file

Additional file 1: **Figure S1.** Kaplan Meier survival curves. CO denotes “control patients who were never admitted to the ICU”, IC means “Patients admitted to the ICU for various reasons other than intoxications” and IX denotes “intoxicated patients admitted to the ICU”. **Figure S2.** Cost per day alive in various intoxication subgroups. Depicted are the median costs (in euro’s and the 75th percentile) per day alive for various types of intoxications (based upon the APACHE IV admission diagnoses for intoxications) for the year prior to ICU admission (2012), the year of ICU admission (2013) and the year after ICU admission (2014). **Figure S3.** Costs and APACHE IV predicted mortality groups. Costs (in euro’s) per day alive for various APACHE IV predicted mortality groups (< 30% predicted mortality, 30–70% predicted mortality, and ≥ 70% predicted mortality). **Table S1.** Definition of intoxication. Here the different admission diagnoses for intoxication categories within the APACHE IV model are described including some examples. **Table S2.** Chronic conditions derived from the Pharmaceutical Cost Groups in the Vektis. Here various chronic conditions and their frequencies within our study population are presented. **Table S3.** Population with one or two chronic conditions. What conditions were present in patients with at least two conditions at the start of our study. **Table S4.** Commonest comorbid conditions and type of intoxication. The commonest comorbidities and conditions present in patients who were admitted to the ICU for an intoxication. (DOCX 226 kb)

## Abbreviations

AIDS: Acquired Immune Deficiency Syndrome
APACHE IV: Acute Physiology and Chronic Health Evaluation IV
CAP: Community Acquired Pneumonia
COPD: Chronic obstructive Pulmonary Disease
CPR: Cardio Pulmonary Resuscitation
CVA: Cerebrovascular Accident
GI: Gastro Intestinal
HIV: Human Immunodeficiency Virus
HRQoL: Health Related Quality of Life
ICU: Intensive Care Unit
IQR: Inter-quartile range
NICE: National Intensive Care Evaluation
OHCA: Out of Hospital Cardiac Arrest
SAH: Subarachnoid Haemorrhage
SAPS II: Simplified Acute Physiology Score II
SD: Standard deviation
SES: Socioeconomic Status
