# Supplementary material for: The healthcare costs of intoxicated patients who survive ICU admission are higher than non-intoxicated ICU patients: a retrospective study combining healthcare insurance data and data from a Dutch national quality registry
Source: BMC Emerg Med. 2019 Jan 11;19:6. doi: 10.1186/s12873-019-0224-7 (PMC6329083; doi:10.1186/s12873-019-0224-7)
Supplement: Supplementary file 1 — Figure S1. Kaplan Meier survival curves. CO denotes “control patients who were never admitted to the ICU”, IC means “Patients admitted to the ICU for various reasons other than intoxications” and IX denotes “intoxicated patients admitted to the ICU”. Figure S2. Cost per day alive in various intoxication subgroups. Depicted are the median costs (in euro’s and the 75th percentile) per day alive for various types of intoxications (based upon the APACHE IV admission diagnoses for intoxications) for the year prior to ICU admission (2012), the year of ICU admission (2013) and the year after ICU admission (2014). Figure S3. Costs and APACHE IV predicted mortality groups. Costs (in euro’s) per day alive for various APACHE IV predicted mortality groups (< 30% predicted mortality, 30–70% predicted mortality, and ≥ 70% predicted mortality). Table S1. Definition of intoxication. Here the different admission diagnoses for intoxication categories within the APACHE IV model are described including some examples. Table S2. Chronic conditions derived from the Pharmaceutical Cost Groups in the Vektis. Here various chronic conditions and their frequencies within our study population are presented. Table S3. Population with one or two chronic conditions. What conditions were present in patients with at least two conditions at the start of our study. Table S4. Commonest comorbid conditions and type of intoxication. The commonest comorbidities and conditions present in patients who were admitted to the ICU for an intoxication. (DOCX 226 kb) [file 12873_2019_224_MOESM1_ESM.docx]

**Additional file 1:**

**The healthcare costs of intoxicated patients who survive ICU admission are higher than non-intoxicated ICU patients: A descriptive study combining healthcare insurance data and data from a Dutch national quality registry**

**Contents:**

**Figure S1:** Kaplan Meier survival curves

**Figure S2**: Cost per day alive in various intoxication subgroups

**Figure S3:** Costs and APACHE IV predicted mortality groups

**Table S1.** Definition of intoxication

**Table S2:** Chronic conditions derived from the Pharmaceutical Cost Groups in the Vektis

**Table S3:** Population with one or two chronic conditions

**Table S4:** Commonest comorbid conditions and type of intoxication

**Figure S1:** Kaplan Meier survival curves


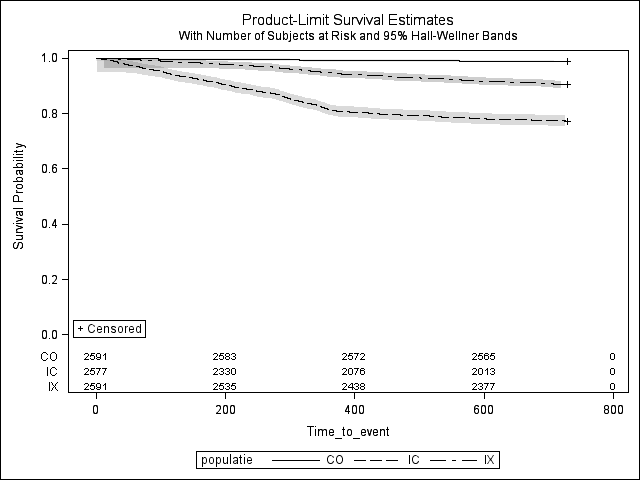


**Legend to Figure S1:** CO denotes “control patients who were never admitted to the ICU”, IC means “Patients admitted to the ICU for various reasons other than intoxications” and IX denotes “intoxicated patients admitted to the ICU”.

**Figure S2**: Cost per day alive in various intoxication subgroups


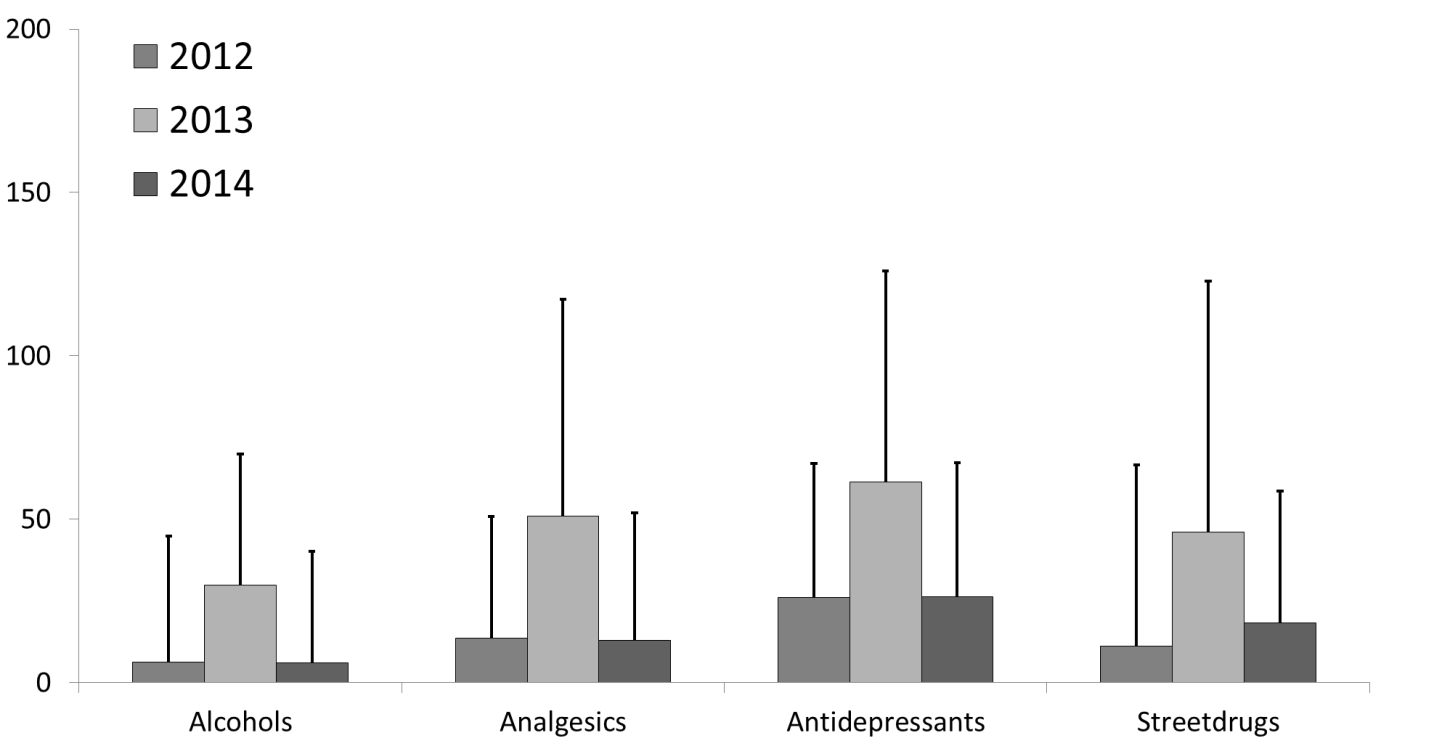


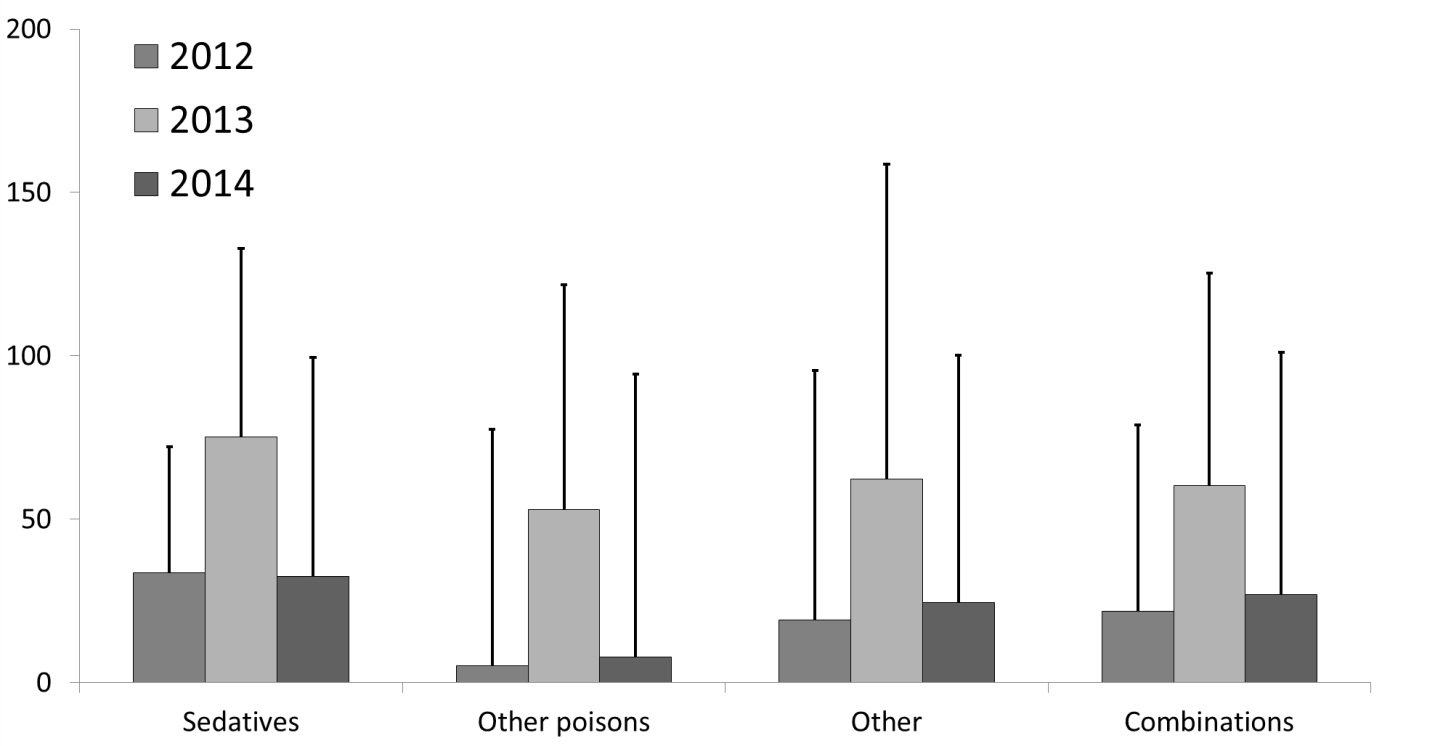


**Legend to Figure S2**: Depicted are the median costs (in euro’s and the 75^th^ percentile) per day alive for various types of intoxications (based upon the APACHE IV admission diagnoses for intoxications) for the year prior to ICU admission (2012), the year of ICU admission (2013) and the year after ICU admission (2014).

**Figure S3:** Costs and APACHE IV predicted mortality groups

**A:** intoxicated patients


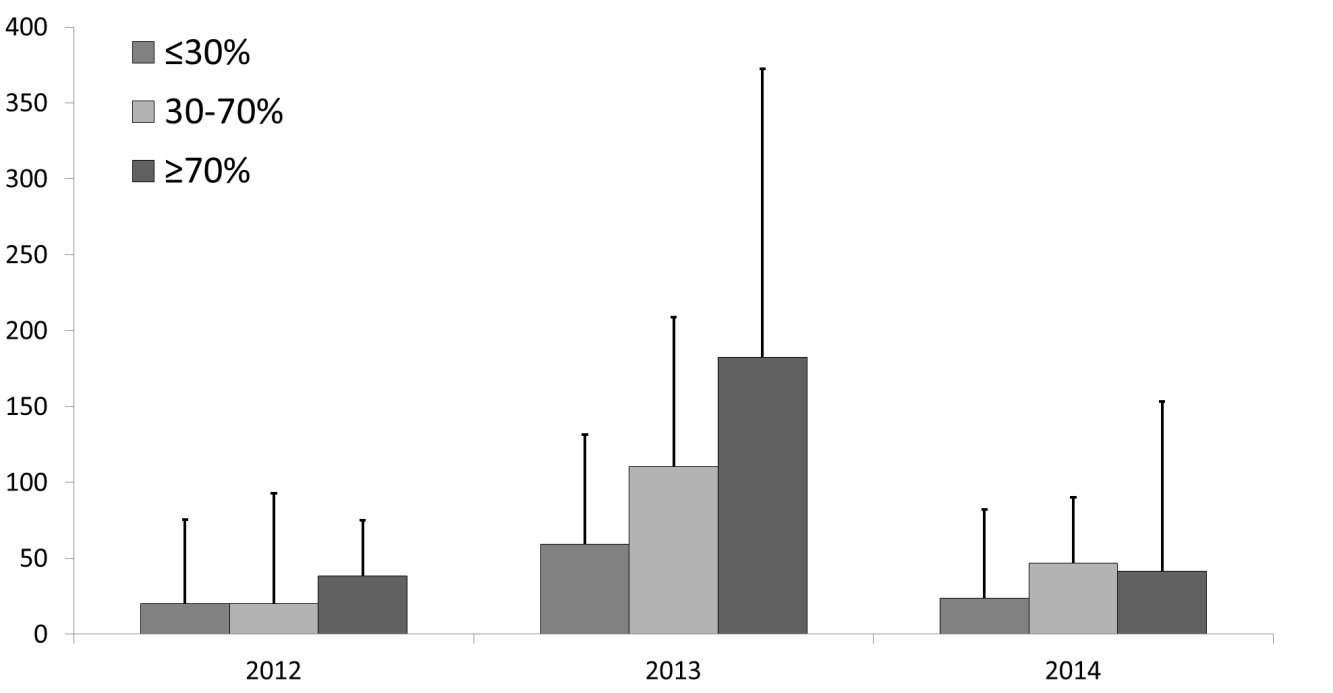


**B:** ICU patients


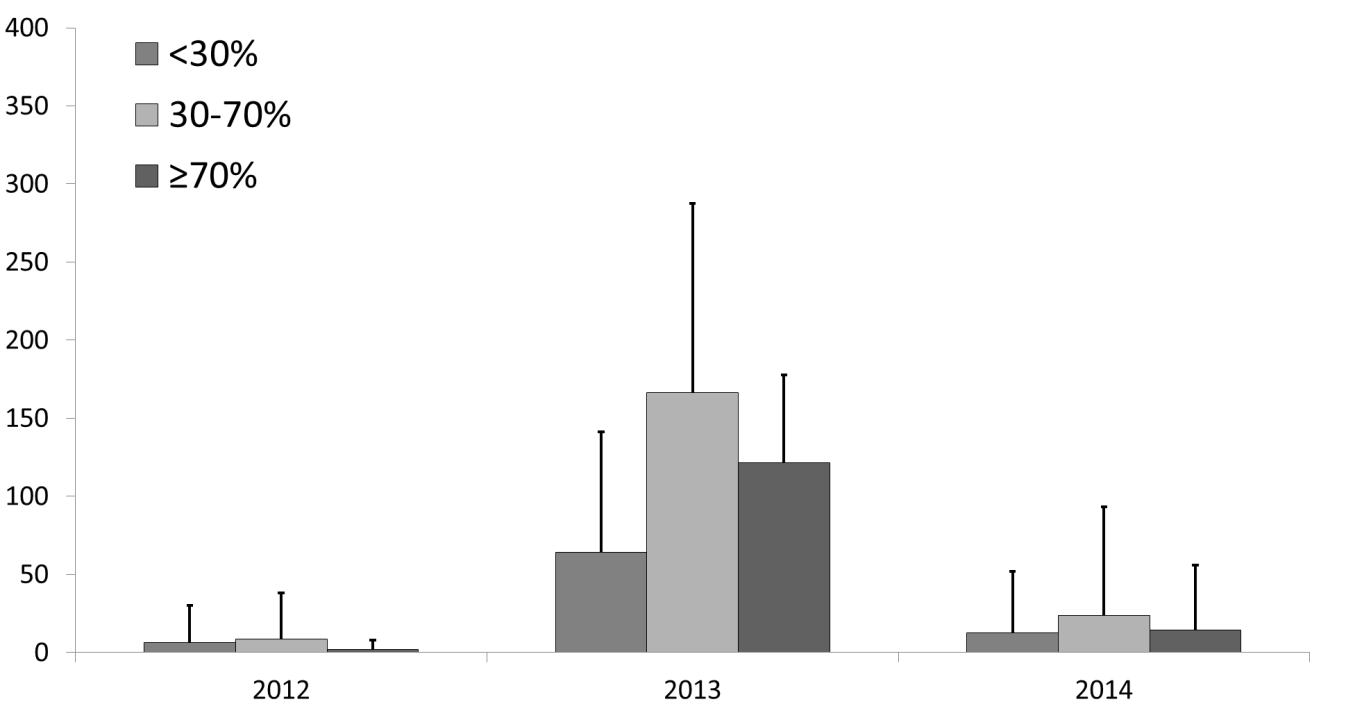


**Legend to Figure S3:** Costs (in euro’s) per day alive for various APACHE IV predicted mortality groups (<30% predicted mortality, 30-70% predicted mortality, and ≥70% predicted mortality).

| **Table S1. Definition of intoxication** | |
| --- | --- |
| Subtype of intoxication | APACHE IV description |
| Alcohol | Overdose, alcohols (e.g. ethanol, methanol, ethylene glycol, etc.) |
| Analgesic | Overdose, analgesic (e.g. aspirin, cetaminophen, paracetamol, etc.) |
| Antidepressant | Overdose, antidepressants (e.g. cyclic, lithium) |
| Street drug | Overdose, street drugs (e.g. opiates, cocaine, amphetamine) |
| Sedatives | Overdose, sedatives, hypnotics, antipsychotics, benzodiazepines |
| Poisoning | Poisoning, carbon monoxide, arsenic, cyanide |
| Other | Overdose with other toxin, poison or drug |
| Combination | Any combination of the above APACHE IV codes |

**Table S2:** Chronic conditions derived from the Pharmaceutical Cost Groups in the Vektis database

| **Chronic conditions 2012** | Intoxication patients  (n = 2591) | Other ICU patients  (n = 2579) | Control population  (n=2591) |
| --- | --- | --- | --- |
| Population with one or more chronic conditions | 1389 (53.6%) | 1038 (40.2%) | 489 (18.9%) |
| Asthma | 126 (4.9%) | 140 (5.4%) | 54 (2.1%) |
| COPD | 100 (3.9%) | 151 (5.9%) | 23 (0.9%) |
| Crohn’s disease | 7 (0.3%) | 7 (0.3%) | 6 (0.2%) |
| Cystic fibrosis / pancreas enzymes | 5 (0.2%) | 14 (0.5%) | 3 (0.1%) |
| Depression | 599 (23.1%) | 188 (7.3%) | 102 (3.9%) |
| Diabetes Mellitus type 1 | 86 (3.3%) | 176 (6.8%) | 26 (1.0%) |
| Diabetes Mellitus type 2 | 60 (2.3%) | 96 (3.7%) | 60 (2.3%) |
| Diseases of the central neurological system | 17 (0.7%) | 20 (0.8%) | 2 (0.1%) |
| Epilepsy | 105 (4.1%) | 68 (2.6%) | 18 (0.7%) |
| Glaucoma | 21 (0.8%) | 26 (1.0%) | 12 (0.5%) |
| Heart diseases | 104 (4.0%) | 201 (7.8%’ | 46 (1.8%) |
| High cholesterol | 151 (5.8%) | 149 (5.8%) | 121 (4.7%) |
| HIV/AIDS | 13 (0.5%) | 11 (0.4%) | 3 (0.1%) |
| Hormone sensitive tumors | 10 (0.4%) | 13 (0.5%) | 9 (0.3%) |
| Kidney diseases | 6 (0.2%) | 32 (1.2%) | 2 (0.1%) |
| Neuropathic pains | 132 (5.1%) | 56 (2.2%) | 11 (0.4%) |
| Parkinson’s disease | 7 (0.3%) | 3 (0.1%) | 5 (0.2%) |
| Psychoses, Alzheimer’s disease and addictions | 357 (13.8%) | 68 (2.6%) | 19 (0.7%) |
| Rheumatism | 9 (0.3%) | 19 (0.7%) | 13 (0.5%) |
| Thyroid diseases | 57 (2.2%) | 80 (3.1%) | 51 (2.0%) |
| Transplantations | 7 (0.3%) | 31 (1.2%) | 8 (0.3%) |

**Table S3:** Population with one or two chronic conditions

| **Population with one chronic condition** | | | | | | |
| --- | --- | --- | --- | --- | --- | --- |
| **Intoxication (n=943)** | | **IC (n=666)** | | **Control (n=404)** | | |
| Depression | 408 | DM type I | 97 | High cholesterol | 90 | |
| Psychoses, Alzheimer’s disease and addictions | 224 | Depression | 94 | Depression | 71 | |
| Asthma | 48 | High cholesterol | 83 | Asthma | 45 | |
| High cholesterol | 44 | Heart diseases | 75 | DM type II | 44 | |
| Neuropathic pains | 40 | Asthma | 63 | Heart diseases | 31 | |
| **Population with 2 chronic conditions** | | | | | | |
| **Intoxication (n=334)** | | **IC (n=264)** | | **Control (n=68)** | | |
| High cholesterol and depression | 30 | DM type II and heart diseases | 17 | High cholesterol and depression | | 8 |
| Epilepsy and psychoses, Alzheimer’s disease and addictions | 26 | High cholesterol and COPD | 15 | Depression and thyroid diseases | | 6 |
| Asthma and depression | 18 | COPD and depression | 15 | High cholesterol and thyroid diseases | | 5 |
| High cholesterol and Psychoses, Alzheimer’s disease and addictions | 16 | COPD and heart diseases | 12 | COPD and heart diseases | | 4 |
| COPD and depression | 15 | Asthma and depression/  DM type I and heart diseases | 11 | High cholesterol and COPD/  High cholesterol and rheumatism/  COPD and DM type II/  DM type II and Psychoses, Alzheimer’s disease and addictions | | 3 |

**Table S4:** Commonest comorbid conditions and type of intoxication

| **Type intoxication** | **Depression**  **(n)** | **Psychoses, Alzheimer’s disease and addictions**  **(n)** | **Neuropathic pains**  **(n)** | **Epilepsy** |
| --- | --- | --- | --- | --- |
| • Alcohol | 27 | 21 | 4 | 9 |
| • Analgesics | 14 | 10 | 9 | 2 |
| • Antidepressant | 124 | 39 | 15 | 11 |
| • Street drug | 30 | 49 | 17 | 8 |
| • Sedatives | 231 | 143 | 49 | 44 |
| • Poisoning | 3 | 0 | 0 | 0 |
| • Other | 70 | 35 | 13 | 14 |
| • Combination | 100 | 60 | 25 | 17 |
